# On a Specific Method for Characterizing Ion Exchange Membranes to Assess Their Functionality in Salinity Gradient Power Generation Through Reverse Electrodialysis, Including the Effect of Temperature

**DOI:** 10.3390/membranes14120255

**Published:** 2024-12-03

**Authors:** Etienne Brauns, Joost Helsen

**Affiliations:** Electrochemistry Excellence Centre (ELEC), Materials & Chemistry Unit, Flemish Institute for Technological Research (VITO), Boeretang 200, 2400 Mol, Belgium; etienne.brauns@gmail.com

**Keywords:** salinity gradient power, reverse electrodialysis, flux, migration coefficient, power density, electrodes, fouling

## Abstract

Salinity gradient power (SGP) by reverse electrodialysis is a promising method for converting SGP into electricity. Instead of the conventional approach of using seawater and freshwater, an alternative method involves using highly concentrated salt solutions (brines) alongside seawater or brackish water. Key factors influencing SGP via reverse electrodialysis (SGP-RE) include the properties of ion exchange membranes, particularly their thickness. This paper outlines a practical experimental set-up that uses both a cation membrane (CM) and an anion membrane (AM). The system is configured with three compartments: two outer compartments filled with highly concentrated brine (HIGH) and a central compartment containing a lower concentration salt solution (LOW), akin to seawater. The compartments are separated by a CM on one side and an AM on the other. The ion transport rate from the HIGH compartments to the central LOW compartment allows for determining the overall ion transport coefficient for thin membranes. Measurements of ion flux and electrochemical voltage under dynamic equilibrium conditions also enable the estimation of the SGP-RE power density (W/m^2^). By controlling the temperature of the HIGH and LOW solutions, this experiment further investigates the significant impact of temperature on ion transport characteristics.

## 1. Introduction

Salinity gradient power (SGP) has been previously proposed by several studies [1,2,3,4]. Two key concepts in SGP are pressure-retarded osmosis [5] and reverse electrodialysis [6,7,8,9,10,11,12,13,14,15]. Significant advancements in reverse electrodialysis research were realized during the European research project REAPower (www.reapower.eu, accessed on 28 November 2024) and documented in [16,17]. More up-to-date details on the ongoing research efforts and applications in the field of reverse electrodialysis (e.g., seawater versus river water or brine versus seawater) are available in [18,19,20,21,22,23], along with the many references cited within these works. The osmotic energy content of a fully saturated sodium chloride (NaCl) solution is estimated to be approximately 7.75 kWh per cubic meter. This solution has an osmotic pressure (https://openmembranedatabase.org/calculators/osmotic-pressure-calculator/, accessed on 14 November 2024) around 280 atm (about 28 MPa), equivalent to the pressure at the base of a hypothetical 2800 m high hydroelectric dam. This energy density of about 7.75 kWh/m^3^ thus corresponds to lifting one cubic meter of water to about 2800 m in height. As one of several potential applications [13,14,18,19,20,21,22,23], this high energy density and the relatively manageable volume requirements of salt solutions indicate that saturated pure NaCl-based SGP-RE (salinity gradient power by reverse electrodialysis) batteries are theoretically feasible, even for local or household use. For reference, a typical household consumes about 10 kWh of electricity daily, making the use of just a few cubic meters of HIGH (saturated NaCl solution) a practical energy storage option. In a hybrid system which includes solar panels or wind turbines, an SGP-RE battery could store surplus daytime electricity by driving ions from LOW back into HIGH via electrodialysis, effectively “recharging” the battery. The SGP-RE battery could then supply power at night or whenever household demand arises. Additionally, a HIGH solution of pure NaCl helps prevent membrane fouling in the SGP-RE stack, thus extending the life of the ion-exchange (IEX) membranes.

**Figure 1:**
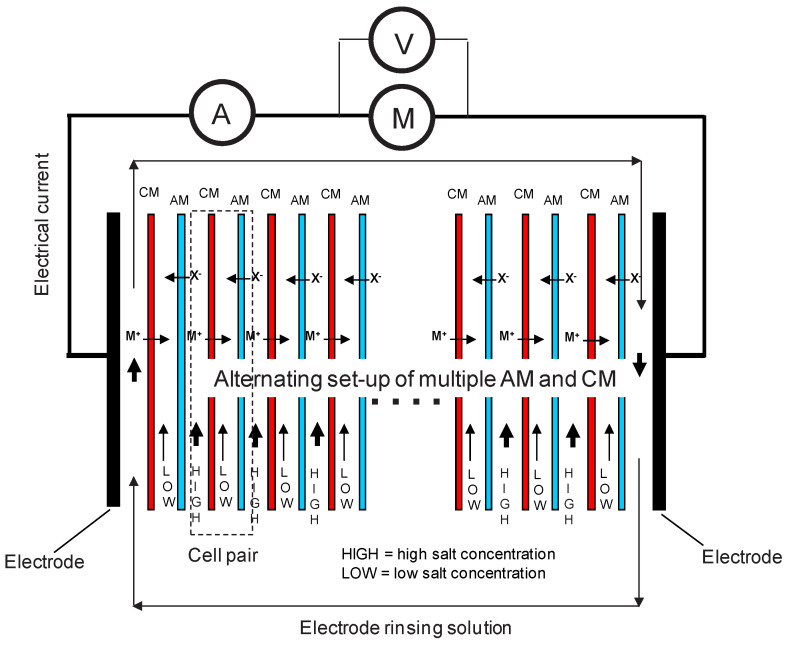
Principle of a complete SGP-RE battery.

Reverse electrodialysis operates by leveraging a significant salinity gradient across ion-selective membranes (Figure 1) using two solutions with markedly different ion concentrations. In this study, these solutions are referred to as HIGH (high concentration) and LOW (low concentration). Cation exchange membranes (CMs) and anion exchange membranes (AMs) are alternately arranged so that HIGH and LOW solutions flow through adjacent compartments. A basic cell pair consists of a HIGH compartment, a CM, a LOW compartment, and an AM in sequence. An SGP-RE stack is created by stacking multiple (N) cell pairs. Across each ion-exchange (IEX) membrane, an electrochemical potential is generated due to the salinity gradient, causing anions to move through the AM from HIGH to LOW compartments and cations through the CM. The electrical power is harvested at the SGP-RE stack’s electrodes.

Figure 1 shows the directional flow of ions in each cell pair, where ion transport corresponds to electrical current and, when combined with the measurable voltage across the membranes, results in the basic power output for each pair. When N cell pairs are connected in series, the total power output is multiplied by N. With a large number of cell pairs (potentially hundreds or thousands in future industrial applications) and thin membranes, the power output (W/m^2^ of membrane or W/m^3^ of SGP-RE stack) could be significantly increased [13,14].

There are two important variants of reverse electrodialysis. The combination of seawater (HIGH) and freshwater (LOW) is often termed “RED” [9,10,11,12], while SGP-RE typically refers to systems using highly concentrated salt solutions (HIGH) with seawater or brackish water (LOW). For instance, the REAPower project adopted the term SGP-RE. SGP-RE can also be integrated into hybrid systems, where HIGH concentrations are achieved by further concentrating brine from seawater desalination units (SWDU), ideally using renewable (solar) energy [6,7]. This hybrid approach could increase potable water production by up to 75% in arid regions [8], adding decisive economic value. The hybrid SGP-RE system may also function as a means of storing electrochemical (osmotic) energy, which can be converted into electricity at night. In remote areas lacking seawater, the SGP-RE battery could be operated using a single salt solution that is re-concentrated using solar energy [13].

Key SGP-RE process parameters, such as membrane thickness and salt solution temperature, were outlined in [13,14]. The high conductivity of the salt solution in both HIGH and LOW compartments offers the potential to enhance power density by reducing membrane thickness, thereby decreasing internal resistance and boosting power output through a higher ion flux across the membranes.

**Figure 2:**
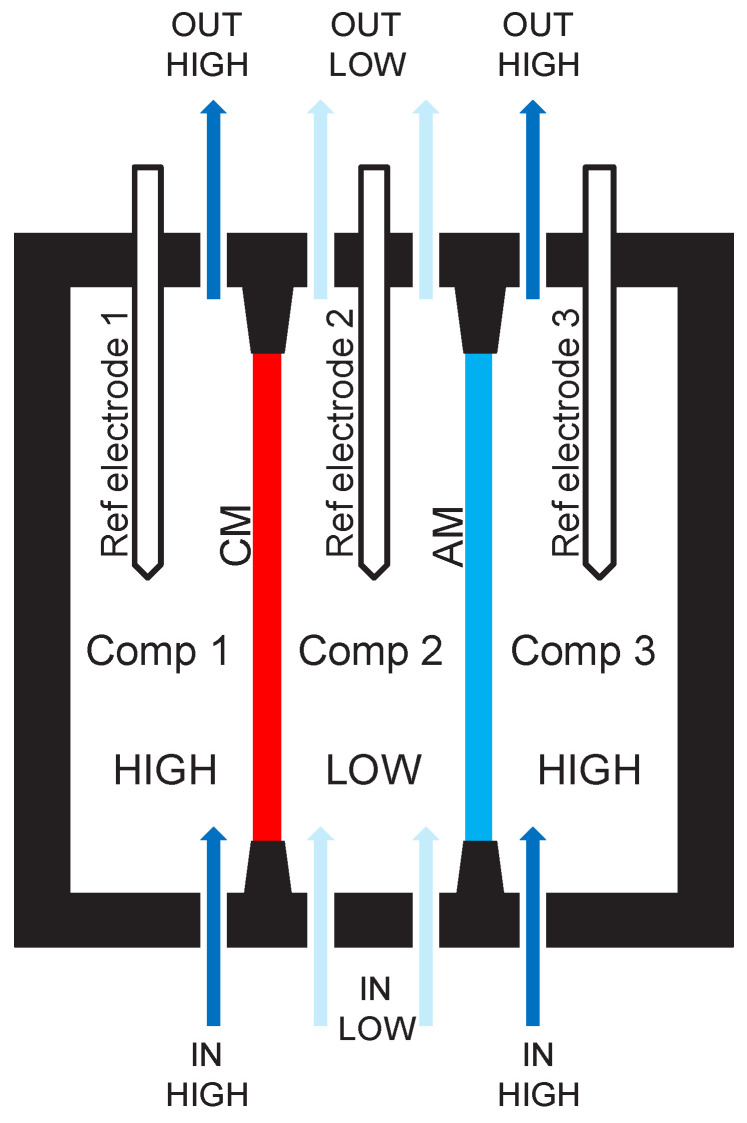
The basic principle of the three-compartment set-up.

This study introduces a three-compartment set-up (Figure 2) to evaluate the overall performance of an AM/CM pair in a practical and technical way. In batch mode experiments, the ion transfer rate (ion flux) from the outer HIGH compartments through the CM and AM into the central LOW compartment is measured by monitoring the increase in the LOW conductivity over time. This rate provides insight into the overall ion migration coefficient of the AM/CM pair, which correlates to ion flux, current density, and power density.

The study reports specifically on the performance of thin CM and AM (10 and 20 µm thickness) and the effect of temperature. The experimental results align with previous theoretical models [13,14], reinforcing the importance of using thin ion exchange membranes in SGP-RE systems. Ion-exchange (IEX) membrane characterization experiments in a complete SGP-RE stack are costly and labor-intensive compared to the simpler three-compartment set-up. This novel method enables a quick and accurate evaluation of cation and anion membrane pairs, rather than measuring membrane properties individually, as well as their combined power density performance in SGP-RE cell pairs. The obtained measurement results therefore form the target for the power density that should be achieved in an actual SGP-RE stack equipped with that membrane pair.

## 2. Materials and Methods

### 2.1. Ion Conductive Membranes

This paper focuses exclusively on homogeneous ion exchange (IEX) membranes. A homogeneous IEX membrane contains fixed ions uniformly distributed throughout its structure as functional side groups along the polymer chain backbone [15]. In cation exchange membranes (CMs), these fixed ions might include groups like SO_3_^−^ or COO^−^, while in anion exchange membranes (AMs), they might include ^+^NH_2_R or ^+^NHR_2_ groups. The inherent uniformity of fixed ion distribution within homogeneous IEX membranes ensures that migrating counterions maintain electro-neutrality within the membrane’s bulk.

In typical electrodialysis applications, IEX membranes function as a “pass-through” medium: an ion_in_ entering one side of the membrane causes another ion_out_ to exit from the opposite side. This ion transport within the membrane can be described as the simultaneous migration of counterions to adjacent fixed ion positions in a process known as “hopping”, which forces the exit of another ion. For single salt solutions such as NaCl, the concentration of counterions in the membrane must balance with the fixed ions to maintain electro-neutrality, resulting in no concentration gradient within the membrane. Thus, the misconception of a linear concentration gradient from the low-concentration to the high-concentration side within the membrane is incorrect. From this perspective, the ion transport mechanism is better described as migration rather than diffusion, as diffusion occurs due to a concentration gradient, which does not exist in the IEX membrane bulk.

Key membrane properties, such as permselectivity, migration coefficient, and membrane thickness, are critical factors influencing the power output of SGP-RE batteries. Although direct measurements on a complete SGP-RE stack are very important, they are labor-intensive and resource-demanding due to the need to assemble a full lab-scale stack with multiple cell pairs and appropriate electrodes. Therefore, this paper proposes a practical and alternative method using a three-compartment set-up. This set-up allows for initial, practical insights into the overall SGP-RE performance of CMs and AMs in a cell pair before conducting more detailed tests in a fully assembled SGP-RE stack.

Previous studies [13,14] have emphasized the importance of thin membranes for achieving high SGP-RE power density (W/m^2^). While ultra-thin IEX membranes (10 µm or thinner) are not yet available on an industrial scale, FuMA-Tech (Bietigheim-Bissingen, Germany) provided membrane samples with a nominal thickness of 20 µm for exploratory tests. The experiments described in this paper aimed to assess the impact of thin membranes on ion migration rates and power output estimates in an SGP-RE stack. Three thin homogeneous membranes were tested: FKS20 (CM), FKE20 (CM), and FAS20 (AM). The thickness of these membranes varied slightly: FAS20 ranged from 17 µm to 23 µm, FKS20 from 19 µm to 21 µm, and FKE20 from 16 µm to 23 µm. IEX characteristics of these membranes can be found at the FuMA-Tech website: https://www.fumatech.com/en/ (accessed on 14 November 2024). Two samples of each membrane type were tested in the three-compartment set-up, and the average values were calculated. Two CM/AM combinations were tested: FKS20/FAS20 and FKE20/FAS20.

### 2.2. Pragmatic Principle of a Three-Compartment Set-Up and Data Acquisition

A set-up was constructed, as shown schematically in Figure 2, consisting of three compartments:

- Compartment 1 is separated from compartment 2 by a cation exchange membrane (CM).
- Compartment 2 is separated from compartment 3 by an anion exchange membrane (AM).

The HIGH solution is circulated through compartment 1, with the outlet (HIGH_OUT) from compartment 1 connected to the inlet (HIGH_IN) of compartment 3. The HIGH_OUT of compartment 3 is then connected back to the pump, creating a continuous recycling loop of the HIGH solution between compartments 1 and 3. Similarly, the LOW solution is pumped into compartment 2, with the outlet (LOW_OUT) recycled back to the inlet (LOW_IN), enabling a batch experiment for both the HIGH and LOW solutions. This configuration establishes a salinity gradient across the membranes due to the difference in concentrations between the HIGH and LOW solutions, driving ion migration through the ion-exchange membranes. Over time, the concentration of cations and anions in the LOW solution increases while the concentration in the HIGH solution decreases, allowing for the measurement of overall ion-migration kinetics for a given pair of CMs and AMs. The three-compartment set-up is an adequate method for characterizing SGP-RE performance, especially when using thin membranes.

In this set-up, the CM and AM samples have an effective exposure diameter of 5 cm, while each compartment has a width of about 6 cm. A reference electrode is positioned in the center of each compartment.

During the experiments, NaCl solutions were used with concentrations of 5 M for the HIGH solution and 0.5 M for the LOW solution. The volume of the HIGH solution was 5 L, and the LOW solution volume was 1.2 L, resulting in a ratio of the number of ions in HIGH to LOW of 41.7. This large ratio provides a significant HIGH buffer effect, meaning the concentration in the LOW solution increases noticeably during the experiment, while the concentration in the HIGH solution decreases only slightly. The salinity difference across the membranes creates an electrochemical potential difference. The batch-type experiment was performed at appropriate flow rates, typically 900 mL per minute, for both HIGH and LOW compartments. As discussed in Section 3, the experimental results indicate that the chosen flow rates were sufficiently high to prevent an excessively low residence time of the HIGH or LOW solutions, ensuring effective ion transport through the IEX membranes. The temperatures of the HIGH and LOW solutions were regulated using a thermostatic bath with heat exchange to maintain consistent temperatures for both solutions.

A three-compartment set-up, as described here, is helpful for the measurement of ion migration in SGP-RE systems. A three-compartment set-up allows Na^+^ to migrate from compartment 1 to 2 and Cl^−^ from compartment 3 to 2, maintaining electro-neutrality and enabling continuous ion transport until equilibrium is reached, following the principle of thermodynamic mixing of salt solutions with different concentrations. In contrast, a two-compartment set-up (one HIGH and one LOW compartment with a single membrane) would only allow one type of ion to migrate, leading to a rapid halt in ion transfer. Trying to measure an SGP-RE-related ion migration in one type of IEX membrane on the basis of a two-compartment set-up is thus not possible.

A conductivity sensor was installed in the LOW solution’s recycling stream to track the increasing concentration over time. The conductivity meter was calibrated using standard NaCl solutions, creating a conductivity–concentration calibration curve. The set-up also included a thermostatic control system to maintain the temperature of both HIGH and LOW solutions within ±0.2 °C of the set points. All measurements were logged throughout the experiments.

It is important to note that in SGP-RE, the main transport mechanism is ion migration through the IEX membranes, while water transport due to osmotic and electro-osmotic forces is minimal, as explained hereafter. Indeed, after several hours of operation, only about 5% of water transport was detected, and in the time frame of one hour (relevant to the data analyzed), water transport accounted for just 1.5%, making it negligible for the calculations. In a real SGP-RE stack, which operates continuously with a limited residence time [13], water transport would have even less significance.

### 2.3. Data Modeling and Processing

In this study, it is assumed that during the experiment using the three-compartment set-up, a dynamic equilibrium is maintained at all times, governed by the two membranes (CM and AM). These membranes do not exhibit identical ion transport rates for cations and anions, nor are they perfectly or equally permselective. As a result, the transport of cations from compartments 1 to 2 and anions from compartments 3 to 2 self-regulates based on the requirement of electro-neutrality in the HIGH and LOW solutions throughout the experiment. The membranes remain “tuned” to this equilibrium.

Equation (1), derived in [14] from the Nernst–Planck equation, is assumed to closely approximate the real ion migration transport phenomena through the membranes, as was the case in [14]. The derivation and details of this equation are available in [14]:

(1) $$J=D_{memb}\cdot \frac{F\cdot c_{memb}\cdot z}{R\cdot T}\cdot \frac{\Delta E}{W_{memb}}$$

where the following variables are defined

*J* = flux [mol/(m^2^.s)];

*D_memb_* = ion migration coefficient [m^2^/s];

*c_memb_* = fixed ion concentration [M];

*W_memb_* = membrane thickness (m);

Δ*E* = potential drop across the membrane (V);

*F* = Faraday number [96,485.34 C/mol];

*R* = gas constant [8.3144621 J/(mol·K)];

*T* = temperature [K];

*z* = ion valence (1 for NaCl experiments).

In [13], Equation (2) was presented:

(2) $$\Delta E=\left(t_{m,cou}-t_{m,co}\right)\cdot \frac{R\cdot T}{F}\cdot \ln\left(\frac{a_{X,HIGH}}{a_{X,LOW}}\right)=\alpha \cdot \frac{R\cdot T}{F}\cdot \ln\left(\frac{a_{X,HIGH}}{a_{X,LOW}}\right)$$

where the following variables are defined

*t_m_*_,*cou*_ = transport number of the counterion in the membrane;

*t_m_*_,*co*_ = transport number of the co-ion in the membrane;

*a_X_*_,*HIGH*_ = activity of ion X in the HIGH solution;

*a_X_*_,*LOW*_ = activity of ion X in the LOW solution;

*α* = membrane permselectivity.

Strathmann [15] suggests that “to a first approximation, the membrane potential is identical to the measured membrane potential”, implying that the potential drop Δ*E* measured across the membrane corresponds to the value in Equation (1). The measured potential includes the effects of ion activity and membrane permselectivity.

By logging the concentration changes in the LOW solution over time, the global ion transfer rate (flux J) can be calculated. The experimentally obtained flux value *J* can then be substituted into Equation (1) to estimate the ion migration coefficient *D_memb_*. However, this approach has limitations: since the experiment measures both the AM and CM together, it provides the overall performance, not the individual contributions of each membrane. To isolate the properties of one membrane, future research could pair it with a membrane having significantly higher transport properties, allowing the target membrane to dictate the ion transport rate. Despite this limitation, the method has clear advantages. A functional SGP-RE cell always consists of both a CM and an AM, and this set-up provides a relevant means to assess the performance of a cell pair. The power output of an SGP-RE battery stack, consisting of multiple cell pairs in series, scales with the number of pairs. Therefore, this method offers a useful first look at SGP-RE performance for specific membrane sets. The proposed approach could serve as a highly relevant technical test that might be standardized. Alternatively, it could be enhanced by incorporating, for example, ion chemical analysis to gain deeper insights into the performance of individual membranes.

Equation (1) can be rewritten as

(3) $$D_{memb}=\frac{J\cdot R\cdot T\cdot W_{memb}}{F\cdot c_{memb}\cdot z\cdot \Delta E}$$

where Δ*E* represents the average potential across both membranes:

(4) $$\Delta E=\frac{{\Delta E}_{1,2}+{\Delta E}_{2,3}}{2}$$

With Δ*E*_1,2_ and Δ*E*_2,3_ representing the potential difference between reference electrodes 1 and 2 and 2 and 3, respectively. Throughout the experiments, the value of Δ*E*_1,2_ + Δ*E*_2,3_ consistently matched the value of Δ*E*_1,3_.

Temperature plays a crucial role in determining *D_memb_* and ion flux, since higher temperatures lead to faster ion migration. Experiments were conducted at 25 °C, 30 °C, 35 °C, and 40 °C, with each membrane pair tested twice at each temperature. New membranes were mounted for each test (after the typical preconditioning treatment in a salt solution, as required for fresh IEX membranes).

**Figure 3:**
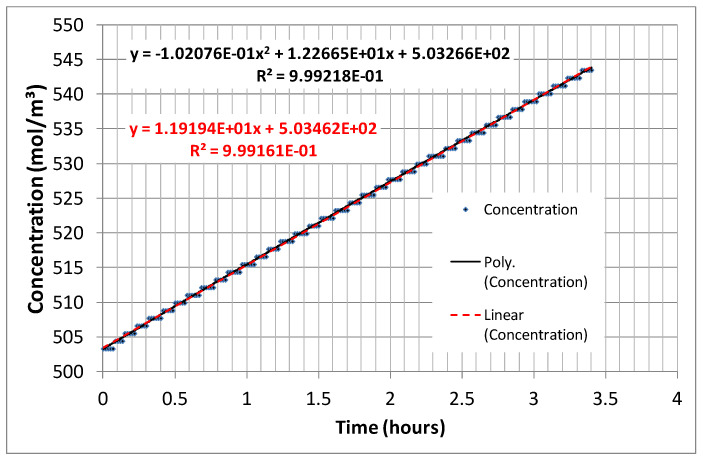
Typical concentration trend of the LOW solution in time.

The conductivity of the LOW solution was continuously logged at one-minute intervals, providing sufficient resolution, as seen in Figure 3. NaCl concentration was calculated from these conductivity measurements using a calibration curve. The stepwise aspect of the measured data is linked to the digital output resolution of the conductivity measurement device. The data were fitted using both linear and second-degree polynomial regressions, with the latter offering a slightly better fit (correlation coefficient > 0.999).

The derivative of the second-degree regression equation expressing conc = f(t), as presented in Figure 3, can be calculated and used in order to estimate the value of d(conc)/dt (slope of the measured conc = f(t)) data and therefore also the value of the flux *J*). Upon knowing all the (average) values within Equation (3), the value of *D_memb_* can then be calculated approximately.

## 3. Results and Discussion

The *D_memb_* values were extracted at two time points: t_1_ = 0 h and t_2_ = 1 h. Table 1 presents the results for the membrane combinations FKS20/FAS20 and FKE20/FAS20. The *D_memb_* values at t_1_ and t_2_ are very close to each other, and for practical purposes (as in a standardized technical test), the mean value is considered representative of *D_memb_*. The strong correlation between the experimental results (as shown later) and theoretical model values [13,14] supports the selection of t_1_ = 0 h and t_2_ = 1 h as appropriate. Additionally, Figure 3 shows a high coefficient of determination, R^2^ = 0.99916, for the linear regression, further confirming the suitability of selecting t_1_ = 0 h and t_2_ = 1 h for this technical test. Accordingly, the test duration can be limited to just over 1 h.

**Figure 4:**
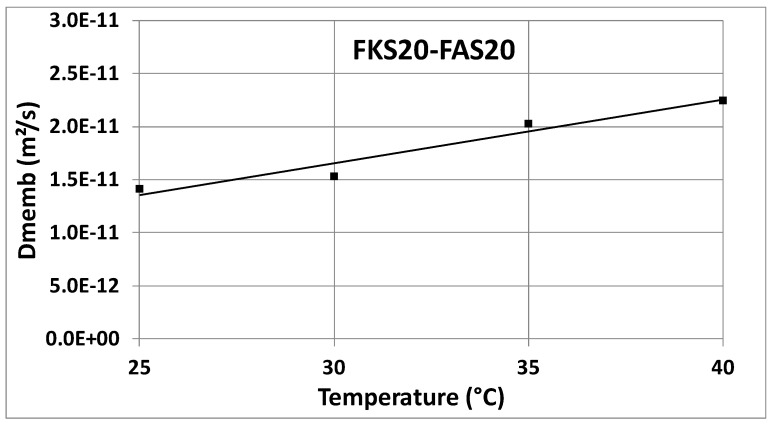
*D_memb_* values versus temperature for the FKS20/FAS20 combination.

**Figure 5:**
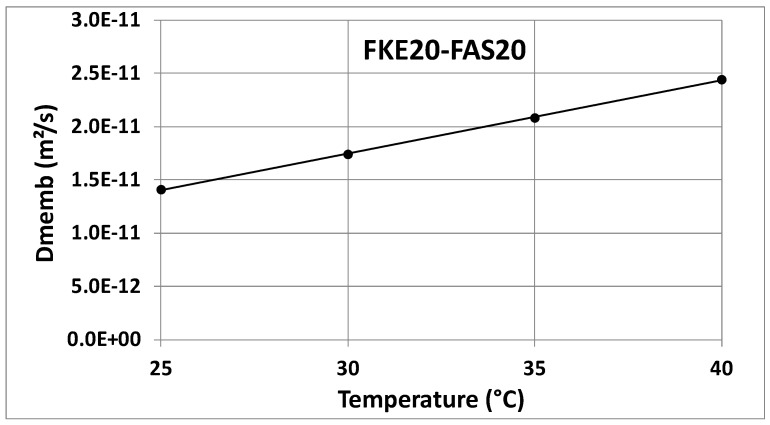
*D_memb_* values versus temperature for the FKE20/FAS20 combination.

Figure 4 and Figure 5 show the *D_memb_* values as a function of temperature, with data from Table 1 graphically represented. The consistency of these values across both combinations (FKS20/FAS20 and FKE20/FAS20) highlights the reliability of this pragmatic approach. This method can be regarded as a promising new technical characterization technique, particularly valuable during the development of thin IEX membranes. It can aid in the design and market introduction of specialized membranes within the SGP-RE field, potentially improving SGP-RE battery output.

The experiments also provided data on

- Ion flux, which corresponds to current density when multiplied by the Faraday constant.
- The voltage across the membranes, used in Equation (3).

Using these data, an estimate of the SGP-RE power density for a set of membranes can be made by multiplying the current density by the mean voltage (as per Equation (4)). As explained in [14], when an electrical load with impedance equal to the internal impedance of the SGP-RE battery is connected, 50% of the power generated by the cell pair is available for external use, while the other 50% is consumed by the internal resistance of the battery. Table 2 and Table 3 present the derived power density values based on experimental data, showing the 50% usable electric power for an external load, along with the corresponding current densities. It is important to note that these power density values are indicative, as actual SGP-RE battery power densities must be determined from a real SGP-RE stack. This method provides an initial indication of the SGP-RE performance of a set of CMs and AMs using only small samples, whereas real SGP-RE experiments require larger membrane areas and more assembly time due to the need for multiple cell pairs and electrodes (anode and cathode set-up).

**Figure 6:**
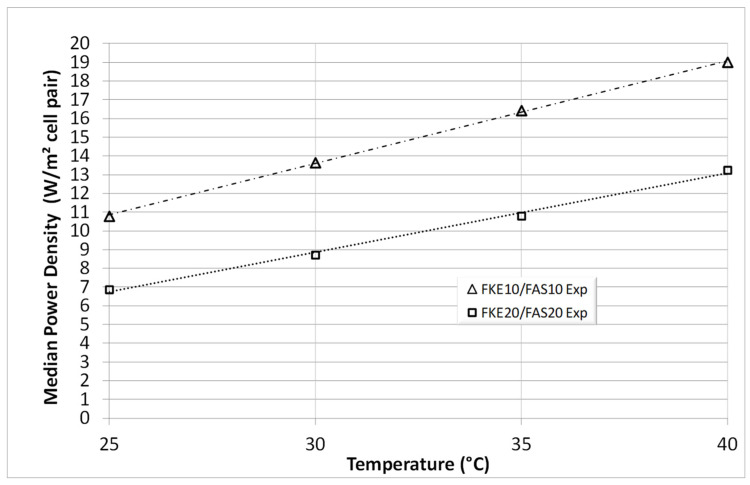
Calculated mean power density versus temperature for the experimental 10 µm FKS/FKE membranes.

**Figure 7:**
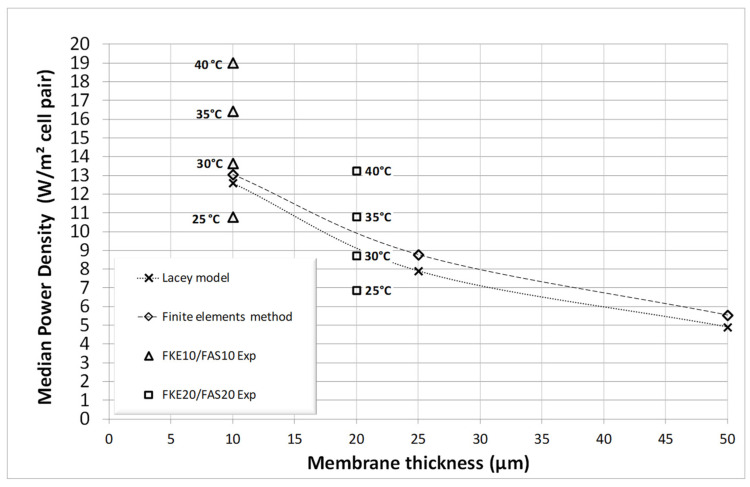
Comparison of calculated mean power density with calculated data from [13] and Figure 10 in [14], both @30 °C.

Figure 6 provides a graphical comparison of the power density values from Table 2 and Table 3 in relation to temperature, illustrating the significant impact of temperature on SGP-RE output [13,14]. Additionally, for comparison, the power density data were implemented into Figure 10 in [14], @30 °C, leading to Figure 7. As the focus of this publication is on “thin” membranes, the x-axis in Figure 7 is limited to 50 µm to emphasize power densities for membranes with a nominal thickness of 20 µm, specifically the FKS20, FKE20, and FAS20 membranes. Figure 7 shows that experimental data from the three-compartment set-up align well with theoretical predictions (based on the Lacey model and finite element calculations [13,14]).

Furthermore, additional experimental membranes with a nominal thickness of 10 µm were later obtained from Fumatech and tested similarly. Results for the median power output are presented in Figure 6 and Figure 7, once again demonstrating the consistency of the three-compartment approach and the significance of membrane thickness on SGP-RE power output. The importance of higher HIGH and LOW temperatures is also evident from these figures.

The theoretical “Lacey model” data points in Figure 7, shown with the dotted regression line, are derived from calculations @30 °C using the Lacey model in TK-Solver [13]. Similarly, the theoretical “Finite Element Method” (FEM) data points, connected by the dashed regression line, are calculated @30 °C using FEM with Comsol Multiphysics [14]. It is clear that the experimental data points in Figure 7, obtained through three-chamber measurements @30 °C for the FKE/FAS membrane pairs with thicknesses of 10 µm and 20 µm, align closely with the theoretical lines @30 °C generated by these two distinct models. The extra experimental data at other temperatures in Figure 7 and obtained in particular at 40 °C for FKE/FAS membranes with a 10 µm thickness highlight the possibility of targeting significantly higher power density outputs in SGP-RE stacks, approaching 20 W/m^2^ per cell pair in that case. These findings should encourage further exploration into the potential of even thinner IEX membranes, which can be readily characterized in a set-up similar to the one proposed here. In this context, effective thin-film coating methods on porous supports, the latter of which would also serve as spacer materials (akin to TFC techniques in reverse osmosis membranes), within an SGP-RE stack could be explored.

These findings confirm the potential of the three-compartment set-up, although it should still be viewed as a technical method providing preliminary data. When performed under controlled conditions (e.g., temperature, flow rate, LOW, and HIGH volume), the test allows for a comparative evaluation of membrane pairs regarding their SGP-RE performance potential.

It is worth noting that the lower power density output observed during the testing of laboratory SGP-RE stacks may, for instance, be attributed to the suboptimal performance of the electrode set-up in the SGP-RE stack. If this is the case, Figure 7 highlights the need to optimize the electrode configuration. Alternatively, other negative factors in the stack design may require further investigation through additional research. The data in Figure 7 suggests that the power density of an SGP-RE stack should be higher. Optimal power output is also associated with achieving impedance matching between the N cell pairs and the transfer of energy to the connected peripheral components, including the SGP-RE stack electrodes. In general, an output system A can only transfer energy in an optimal way into an input system B if the output impedance of system A matches the input impedance of system B. In that respect, a literature search on the basis of “Electrode design for reverse electrodialysis”, “Electrode phenomena modelling in salinity gradient power”, “Reverse electrodialysis membrane electrode assembly and electrode efficiency”, and “Ion transport in reverse electrodialysis electrodes” showed that only very limited and rather unspecific information is available at the moment (2024) in this specific research domain. This leaves room for further research and dedicated optimization of the (electrode) design of an SGP-RE stack, targeting the power density data as illustrated in Figure 7.

Additionally, the three-compartment set-up discussed in this publication could be utilized to study the effects of IEX membrane fouling. This could be performed either by measuring membranes that have already fouled or by observing the progression of membrane fouling within the three-compartment set-up itself. Fouling involves other ions (bivalent), biofouling, and other possible fouling phenomena [24,25].

## 4. Conclusions

A promising alternative method for measuring the SGP-RE performance of CM/AM sets has been introduced. This approach reveals the effect of membrane thickness and salt solution temperature more quickly and provides insights into the combined migration coefficient of the two membranes. It also allows for an initial estimate of the SGP-RE current density and power density. The method is fast, straightforward, and offers valuable preliminary data on the SGP-RE performance of ion-conductive membranes.

## Figures and Tables

**Table 1:** Experimental results for the combinations of FKS20/FAS20 and FKE20/FAS20.

	FKS20-FAS20			FKE20-FAS20		
	t_1_	t_2_	Mean Value at t_1_&t_2_	t_1_	t_2_	Mean Value at t_1_&t_2_
Temp.	*D_memb_* (m^2^/s)	*D_memb_* (m^2^/s)	*D_memb_* (m^2^/s)	*D_memb_* (m^2^/s)	*D_memb_* (m^2^/s)	*D_memb_* (m^2^/s)
25 °C	1.402 × 10^−11^	1.430 × 10^−11^	1.416 × 10^−11^	1.373 × 10^−11^	1.440 × 10^−11^	1.407 × 10^−11^
30 °C	1.506 × 10^−11^	1.558 × 10^−11^	1.532 × 10^−11^	1.703 × 10^−11^	1.772 × 10^−11^	1.738 × 10^−11^
35 °C	1.980 × 10^−11^	2.077 × 10^−11^	2.028 × 10^−11^	2.042 × 10^−11^	2.118 × 10^−11^	2.080 × 10^−11^
40 °C	2.182 × 10^−11^	2.313 × 10^−11^	2.248 × 10^−11^	2.371 × 10^−11^	2.508 × 10^−11^	2.440 × 10^−11^

**Table 2:** Power densities for the combination of FKS20 and FAS20.

FKS20_FAS20	A	B	Mean of A&B	C	D	Mean of C&D
	Power Dens. @0 h	Power Dens. @1 h	Power Dens.	Current Dens. @0 h	Current Dens. @1 h	Current Dens.
	(W/m^2^)	(W/m^2^)	(W/m^2^)	(A/m^2^)	(A/m^2^)	(A/m^2^)
25 °C	7.38	7.32	7.35	163.5	164.4	164.0
30 °C	7.93	8.22	8.08	173.9	179.9	176.9
35 °C	10.62	10.78	10.70	228.5	235.7	232.1
40 °C	11.83	12.13	11.98	251.2	261.8	256.5

**Table 3:** Power densities for the combination of FKE20 and FAS20.

FKE_FAS	A	B	Mean of A&B	C	D	Mean of C&D
	Power Dens. @0 h	Power Dens. @1 h	Power Dens.	Current Dens. @0 h	Current Dens. @1 h	Current Dens.
	(W/m^2^)	(W/m^2^)	(W/m^2^)	(A/m^2^)	(A/m^2^)	(A/m^2^)
25 °C	6.79	6.94	6.87	156.0	161.6	158.8
30 °C	8.65	8.78	8.72	194.5	199.7	197.1
35 °C	10.73	10.86	10.80	235.2	240.9	238.1
40 °C	13.13	13.37	13.25	280.3	290.9	285.6

## Data Availability

The raw data supporting the conclusions of this article will be made available by the authors on request.
